# Defining County-Level Terrestrial Rabies Freedom Using the US National Rabies Surveillance System: Surveillance Data Analysis

**DOI:** 10.2196/43061

**Published:** 2023-04-07

**Authors:** Amber Kunkel, Gabriella Veytsel, Sarah Bonaparte, Haillie Meek, Xiaoyue Ma, Amy J Davis, Jesse Bonwitt, Ryan M Wallace

**Affiliations:** 1 Division of High-Consequence Pathogens and Pathology National Center for Emerging & Zoonotic Infectious Diseases US Centers for Disease Control and Prevention Atlanta, GA United States; 2 Epidemic Intelligence Service US Centers for Disease Control and Prevention Atlanta, GA United States; 3 Epidemiology Elective Program US Centers for Disease Control and Prevention Atlanta, GA United States; 4 Wildlife Services Animal and Plant Health Inspection Service United States Department of Agriculture Fort Collins, CO United States

**Keywords:** rabies, surveillance, zoonoses, zoonosis, model, disease spread, infection spread, animal, predict, public health

## Abstract

**Background:**

Rabies is a deadly zoonotic disease with nearly 100% fatality rate. In the United States, rabies virus persists in wildlife reservoirs, with occasional spillover into humans and domestic animals. The distribution of reservoir hosts in US counties plays an important role in public health decision-making, including the recommendation of lifesaving postexposure prophylaxis upon suspected rabies exposures. Furthermore, in surveillance data, it is difficult to discern whether counties have no cases reported because rabies was not present or because counties have an unreported rabies presence. These epizootics are monitored by the National Rabies Surveillance System (NRSS), to which approximately 130 state public health, agriculture, and academic laboratories report animal rabies testing statistics. Historically, the NRSS classifies US counties as free from terrestrial rabies if, over the previous 5 years, they and any adjacent counties did not report any rabies cases and they tested ≥15 reservoir animals or 30 domestic animals.

**Objective:**

This study aimed to describe and evaluate the historical NRSS rabies-free county definition, review possibilities for improving this definition, and develop a model to achieve more precise estimates of the probability of terrestrial rabies freedom and the number of reported county-level terrestrial rabies cases.

**Methods:**

Data submitted to the NRSS by state and territorial public health departments and the US Department of Agriculture Wildlife Services were analyzed to evaluate the historical rabies-free definition. A zero-inflated negative binomial model created county-level predictions of the probability of rabies freedom and the expected number of rabies cases reported. Data analyzed were from all animals submitted for laboratory diagnosis of rabies in the United States from 1995 to 2020 in skunk and raccoon reservoir territories, excluding bats and bat variants.

**Results:**

We analyzed data from 14,642 and 30,120 county-years in the raccoon and skunk reservoir territories, respectively. Only 0.85% (9/1065) raccoon county-years and 0.79% (27/3411) skunk county-years that met the historical rabies-free criteria reported a case in the following year (99.2% negative predictive value for each), of which 2 were attributed to unreported bat variants. County-level model predictions displayed excellent discrimination for detecting zero cases and good estimates of reported cases in the following year. Counties classified as rabies free rarely (36/4476, 0.8%) detected cases in the following year.

**Conclusions:**

This study concludes that the historical rabies freedom definition is a reasonable approach for identifying counties that are truly free from terrestrial raccoon and skunk rabies virus transmission. Gradations of risk can be measured using the rabies prediction model presented in this study. However, even counties with a high probability of rabies freedom should maintain rabies testing capacity, as there are numerous examples of translocations of rabies-infected animals that can cause major changes in the epidemiology of rabies.

## Introduction

### Background

Rabies is a deadly zoonotic disease caused by an RNA virus (rabies virus) in the *Lyssavirus* genus, which causes acute progressive encephalitis in mammals with nearly 100% fatality rate [1,2]. Globally, an estimated 60,000 people die annually from rabies infection, with the highest fatality rates attributable to the dog-mediated rabies virus variant (DMRVV) in African and Asian countries [3]. However, established dog population management and vaccination methods have successfully eliminated DMRVV in most Western countries [4].

With the elimination of DMRVV in the United States, terrestrial mesocarnivores and bats are the remaining rabies reservoirs in the United States [5]. Reported infections in domestic animals have decreased since the implementation of animal control and vaccination programs in the 1940s and 1950s in the United States; however, contact with wildlife and unvaccinated domestic animals can still pose a threat of infection [6,7]. Human rabies deaths in the United States are rare, and rabies pre-exposure prophylaxis and postexposure prophylaxis (PEP) are effective when administered according to the Advisory Committee on Immunization Practices guidance [6,8,9]. Annually, an estimated 55,000 to 60,000 people in the United States receive rabies PEP for suspected rabies exposures at an estimated cost of >US $150 million [6,10]. The distribution of reservoir hosts in a given US county (ie, local administrative unit) can inform rabies risk assessment algorithms to determine if rabies PEP is required following a human exposure, based on the epizootiology of the area where the exposure occurred [11]. Therefore, understanding the geographic and temporal patterns of animal rabies in the United States through routine surveillance is essential for public health interventions such as the recommendation of costly but lifesaving rabies PEP as well as for developing and evaluating wildlife rabies management actions and responding to unexpected rabies occurrences [7].

Rabies reservoirs in the United States include multiple bat species and 5 terrestrial mesocarnivores, including raccoons (*Procyon* *lotor*), skunks (family *Mephitidae*), foxes (*Vulpes* spp and *Urocyon* *cinereoargenteus*), and the small Indian mongoose (*Herpestes* *auropunctatus*) in Puerto Rico [7]. Substantial geographical variation exists in the risk of rabies exposure in the United States owing to the distinct geographic distribution of the different rabies virus variants (RVVs) associated with these terrestrial reservoirs [12]. The eastern raccoon RVV, for example, was first reported in Florida in the 1950s and later spread throughout the east coast following an apparent translocation event [13,14]. Three distinct skunk RVVs exist: south central, north central, and California skunks, named for their geographical locations within the United States. Other regions of the United States, such as the northwestern states of Washington and Oregon, are believed to be free from terrestrial rabies reservoirs. However, bat rabies is present throughout the continental US as it is uniquely unconstrained by the geographic barriers that define the territories of terrestrial reservoirs [5,7,12].

Animal and human rabies are both nationally notifiable conditions in the United States [15,16]. The US Centers for Disease Control and Prevention (CDC) manages the National Rabies Surveillance System (NRSS), which comprises approximately 130 jurisdictional public health, agriculture, and academic laboratories that conduct passive public health surveillance, as well as the US Department of Agriculture National Wildlife Rabies Management Program, which conducts active case surveillance in areas where wildlife vaccinations are distributed [12]. Jurisdictional laboratories conduct diagnostic testing; some perform viral characterization for positive samples; and the US CDC offers laboratory assistance for diagnostics, viral typing, and exposure tracing and assessment. Animal and human rabies case definitions and data elements reportable to the NRSS are defined by the Council of State and Territorial Epidemiologists Animal Rabies and Human Rabies position statements, respectively [17,18].

### Objectives

As the NRSS is largely a passive public health surveillance system, it can be difficult to discern through surveillance data if counties report no animal rabies cases because of the absence of rabies or because of the inability to detect cases. Historically, the NRSS defines counties in the United States as *terrestrial rabies free* if there have been no terrestrial rabies cases reported in that county or any adjacent county for the previous 5 years and if the county has achieved a sufficient level of surveillance testing for rabies over those 5 years [12]. However, no previous attempts have been made to validate or improve this definition since its inception in 2005 [19]. Therefore, the objectives of this analysis were to describe and evaluate the performance of the historical NRSS rabies-free county definition, review the possibilities for improving this definition, and develop a model to achieve more precise estimates of the probability of terrestrial rabies freedom and the number of reported terrestrial rabies cases at the county level.

## Methods

### Domestic Animal Rabies Testing and Reporting in the United States

The NRSS collects data from state public health, agriculture, and academic laboratories that test animal samples for rabies using the direct fluorescent antibody test or other diagnostic methods recommended by the Council for State and Territorial Epidemiologist’s Animal Rabies position statement [17]. Confirmed animal cases are reportable to the state public health officials and notifiable to the CDC [15,17,20]. Recommended data elements included the species, capture location, test date, and RVV (when available) of each animal tested for rabies virus. The NRSS has been relatively unchanged since 1995 in terms of consistent diagnostic approaches, access to testing, and epidemiological circumstances.

The passive public health surveillance system prioritizes the testing of animals involved in human or domestic animal exposures, although specific testing criteria vary by jurisdiction. Passive public health surveillance accounted for 95% of reported rabid animals. Active surveillance is conducted in selected high-priority areas and informs wildlife rabies management such as management of oral rabies vaccination campaigns. Passive public health data and active surveillance data were both included in this analysis.

### Historical Rabies-Free Definition

The historical definition used to classify counties as terrestrial rabies free is as follows [12]:

- No terrestrial rabies cases reported in that county for ≥5 years;
- No terrestrial rabies cases reported in any neighboring counties for ≥5 years; and
- ≥15 surveillance points were tested over the past 5 years, where surveillance points=0.5 × (number of domestic animals) + 1 × (number of reservoir animals tested). Companion animals and livestock (eg, dogs, cats, cattle, and horses) are considered domestic animals. Foxes, raccoons, skunks, and mongooses are considered reservoir animals, regardless of the state or county in which they are found.

### Data Set

This analysis used county-level data from the NRSS from 1995 to 2020 regarding the number and species of animals tested, and positive, for rabies each year (excluding bats) as well as accompanying viral characterization results when available. Bats and bat variants were excluded to focus the analysis on rabies from terrestrial reservoirs; the entire continental United States is considered to be at risk for rabies transmitted by bats. First, we evaluated the historical surveillance definition using measures of sensitivity, specificity, positive predictive value, and negative predictive value. Next, we applied a zero-inflated negative binomial model to describe the contributions of each individual variable involved in the historical definition, evaluate alternative definitions of rabies freedom, and predict the presence or absence and the number of rabies cases that would be reported in a given county in the following year.

All bats submitted for testing, as well as rabies virus–positive terrestrial mammals recorded as having bat RVVs, were excluded from the analysis. As data files for some states before 2014 included inconsistent reporting of negative test results, we included data from each state starting from the year after which <3 in 4 consecutive years contained incomplete rabies test results (1995 for most states; 1998 for Delaware; 2002 for Florida, Mississippi, New Mexico, and Vermont; 2006 for California, Georgia, Iowa, and South Carolina; and 2014 for Oklahoma). Eight remaining state-years had missing county information for >50% of negative results. In these data files, to avoid loss of these data in the analysis, the negative results were assigned to counties using the average distribution from the 4 closest years of complete data. Nineteen state-years had ≤10 samples tested or <50% of samples testing negative, suggesting that negative results had not been fully reported. In these data files, we set the number of negatives equal to the average of the 4 closest years of the complete data. Overall, 6.99% (104/1486) of the state-years of data were removed for missing negatives and 2% (27/1350) had negative results imputed.

The total number and species of animals tested and positive were aggregated by county and year. All terrestrial species were included unless they were noted as having bat RVVs. The historical rabies freedom definition requires data on the detection of rabies in the preceding 5 years. We used the first 8 continuous years of data from each county (regardless of whether any samples were submitted) as a baseline and began predicting the presence or absence of rabies from the 9th year of the data. Thus, our predictions began in 2003 for counties in the states with complete data.

Models were fitted separately for counties in states that were historically designated as raccoon and skunk reservoir territory based on past detections of the respective RVVs, as reported by the NRSS [12]. Counties in states with other terrestrial rabies reservoirs (Arctic fox variant, gray fox variant, and dog-mongoose variant) were excluded to focus on the most common RVVs found in the United States. For the raccoon territory, we first selected all counties that had at least 1 recorded positive case in the data set and were located in states where raccoons were the primary terrestrial reservoir. We then added all counties within 100 km of this area (county centroid-to-centroid) unless the new county was located in a state with a different terrestrial reservoir. We followed the same procedure to define the counties as skunk territories.

For modeling analyses, data were separated into “training” and “validation” data sets. Data from 2018 were used to train the models, whereas data from 2019 and 2020 were used as separate validation data sets. The results of nonparametric analyses are presented for both the training and validation data sets.

### Modeling Approach

We used a zero-inflated negative binomial regression model to describe the presence or absence of terrestrial rabies and the number of reported terrestrial rabies cases per county-year. A zero-inflated negative binomial model is appropriate for count data when there is a greater than expected number of zeros in the data set, and the process generating these zeros is independent of the count process [21,22]. In this case, excess zeros may occur in counties that are truly rabies free, that is, not enzootic for terrestrial RVVs. The counting process represents rabid animal observations in counties where rabies is enzootic. The first process is encoded as a logistic model for rabies freedom (1a) and the second as a negative binomial count model (1b). We refer to the zero-inflated negative binomial model as the rabies prediction model.


Freedom model 1a: logit(*P*(*rabies free*)) = *α_0_* + *α_1_A* + *α_2_B* + *α_3_C* + *α_4_D* **(1)**

Count model 1b: log(*E*(*cases reported*|*not rabies free*)) = *β_0_* + *β_1_A* + *β_2_B* + *β_3_E* **(2)**

*Where:*

*A* = log(number of rabid animals reported in the county of interest in the past year + 0.5) **(3)**

*B* = log(number of rabid animals reported in any neighboring county in the past year + 0.5) **(4)**

*C* = log(years with no rabies reported in the county of interest) [1=case reported in past year, 2=last case reported in the year before last, etc] **(5)**

*D* = log(years with no rabies reported in any neighboring county) [1=case reported in the past year, 2=last case reported in the year before last, etc] **(6)**

*E* = log(surveillance points in the past 5 years + 0.5) **(7)**

The count portion of the model estimates the number of cases that will be reported if rabies is enzootic. The freedom portion of the model estimates the probability that there truly are no cases within the county. From these model outputs, we can derive three outcomes: (1) the probability that the county is rabies free, (2) the probability that the county would observe rabies cases (if present) through their surveillance efforts, and (3) the probability that the county would have cases that would go unreported by surveillance efforts.

Predictor variables for each component model were selected based on the historical rabies-free definition and a consideration of possible causal pathways. For example, surveillance may have a causal effect on the number of rabies cases reported (count model) but not on the underlying endemicity of terrestrial rabies RVVs (freedom model).

We natural log transformed all the variables to provide a better fit. We defined the number of years since rabies was last reported as ranging from 1 (last year) to 9 (cutoff for any >8). Variables for surveillance points and cases reported in the prior year were shifted by 0.5 to avoid zero values in log-transformed variables.

### Model Validation and Comparison

To evaluate the impacts of different surveillance effort definitions, we applied different definitions of surveillance effort to the rabies prediction model: including only domestic or only reservoir animals, adjusting the relative value of surveillance effort by changing the coefficient applied to domestic animals from 0.1 to 1 times that of reservoir animals, applying specific coefficients to different domestic and wild animal species based on their reported test positivity rates, changing the number of years of surveillance effort considered from 1 to 8, and normalizing surveillance effort by county size or population.

Several criteria were used to evaluate different model formulations. First, we calculated the Spearman correlation between the predicted and reported cases each year. Second, we computed the area under the receiver operating characteristic curves (AUCs), treating the observed data in the prediction year as “true” values. We considered two different options as predicted values for AUCs: (1) the model-predicted probability of rabies freedom and (2) the model-predicted probability of reporting 0 cases. As the number of observed cases in the prediction year is not a perfect measure of rabies freedom, we also considered the detection of any rabies cases in the following 5 years as a marker of true rabies presence in a sensitivity analysis.

*P*(*rabies free*) = *expit*(*α_0_* + *α_1_A* + *α_2_B* + *α_3_C* + *α_4_D*) **(8)**

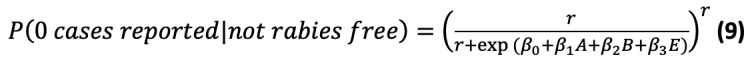



*P(0 cases reported)* = *P(rabies free)* + (1 − *P(rabies free)*) × *P(0 cases reported|not rabies free)* **(10)**

*Where:*

*r*=the negative binomial dispersion parameter

In surveillance data, it is difficult to discern which counties had zero cases reported because rabies was not present versus which counties had unreported rabies. To approximate how well the model captures observing at least 1 case when a county is enzootic, we calculated the AUC by comparing the predicted probability of rabies observing at least 1 positive case if rabies is present in a county (ie, the probability of >0 cases reported from model 1b) with county-level bat observation data in a sensitivity analysis using bat variant data. Rabid bats are considered to be present everywhere in the continental United States, so we hypothesized that counties with a high probability of observing terrestrial rabies would also be more likely to observe rabid bats. However, counties not observing rabid bats would still not be considered “rabies free,” hence their exclusion from model fitting and the main analysis.

### Calculations

All analyses were performed using R [23]. The zero-inflated models were run using the package *pscl* [24].

### Ethical Considerations

These data were reported to the CDC by the State Public Health Departments under the authority of the Council for State and Territorial Epidemiologists, Position Statement 22-ID-06. As this study is a secondary analysis of data, institutional review board approval was not needed.

## Results

### Overview

Our analyses for raccoon territory included data from 1005 counties across 23 states beginning in 2003 for a total of 14,642 county-years. For skunk territory, we included 1799 counties across 25 states beginning in 2003 for a total of 30,120 county-years. Counties from some states were included after 2003, as described in the *Methods* section.

**Figure 1:**
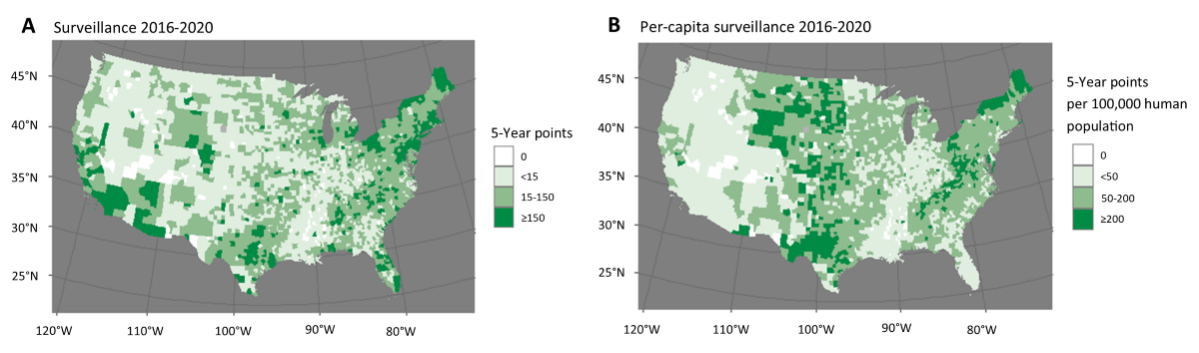
Rabies surveillance points (A) and per-capita rabies surveillance points (B) tested in the contiguous United States during 2016 to 2020 at the county level. Each reservoir animal tested contributes 1 surveillance point, and each domestic animal contributes 0.5 surveillance points.

Of the county-years included for raccoon territory analysis, 57% (8346/14,642) had reported a terrestrial rabies case in the past year, 2% (293/14,642) had not reported a case for exactly 5 years, and 23% (3368/14,642) had not reported a case for >8 years. In skunk territory, only 17% (5120/30,120) of county-years had reported a case in the past year, 3% (904/30,120) had not reported a case for exactly 5 years, and 54% (16,265/30,120) had not reported a case for >8 years. The median number of surveillance points, under the existing NRSS definition, over the preceding 5-year period was 63.0 (IQR 22.0-143.9) in raccoon territory counties and 14.5 (IQR 5.5-39.0) in skunk territory counties. This corresponds to a median of 98.2 (IQR 55.7-153.6) surveillance points per 100,000 people in raccoon territory and 72.9 (IQR 36.2-147.2) per 100,000 people in skunk territory. The overall and per capita surveillance points from 2016 to 2020 are shown in Figure 1.

### Nonparametric Evaluation of Historical Definition

Only 0.85% (9/1065) of counties in raccoon territory and 0.79% (27/3411) of counties in skunk territory had rabies reported in a year for which rabies was predicted to be absent using the historical rabies-free definition from 2003 to 2020 (Table 1). According to the historical definition, counties predicted as rabies free for a given year had no cases reported 99.2% of the time in both raccoon and skunk territories (negative predictive value). Counties predicted to be rabies free for a given year also had a high probability of no cases being reported throughout the following 5 years (97.8% in raccoon territory and 94.9% in skunk territory). Only a small proportion of counties with zero rabies cases reported in a given year met the historical rabies-free criteria (16.6% in raccoon territory and 13.5% in skunk territory).

We performed a deeper investigation into the 9 counties in raccoon territory where rabies was reported in a year for which rabies was predicted to be absent. Of these 9 detections, 2 (22%) were attributed to bat variants that had not been reported to the CDC and 1 (11%) was associated with a data entry error in the state submission file. Of the remainder, 3 were associated with a rabies incursion into northeastern Ohio in 2004 and 1 with a translocated rabid cat [25]. No explanation was found for the remaining 2 counties. Therefore, only 56% (5/9) of detections in raccoon territory were falsely predicted to be rabies free.

**Table 1:** Nonparametric evaluation of historical rabies freedom definition in counties in both skunk and raccoon territories.

Reservoir and county-level rabies prediction (historical rabies-free definition)			Rabies reported in county in the next 1 year				Rabies reported in county in the next 5 years		
		Yes		No	Total	Yes		No	Total
**Raccoon**									
	Present, n	8264		5313	13,577	7756		2090	9846
	Free, n	9		1056	1065	17		759	776
	P(predicted present|reported)	99.9%		99.9%	99.9%	99.8%		99.8%	99.8%
	P(predicted free|not reported)	16.6%		16.6%	16.6%	26.6%		26.6%	26.6%
	P(reported|predicted present)	60.9%		60.9%	60.9%	78.8%		78.8%	78.8%
	P(not reported|predicted free)	99.2%		99.2%	99.2%	97.8%		97.8%	97.8%
**Skunk**									
	Present, n	4944		21,765	26,709	8298		12,074	20,372
	Free, n	27		3384	3411	131		2421	2552
	P(predicted present|reported)	99.5%		99.5%	99.5%	98.4%		98.4%	98.4%
	P(predicted free|not reported)	13.5%		13.5%	13.5%	16.7%		16.7%	16.7%
	P(reported|predicted present)	18.5%		18.5%	18.5%	40.7%		40.7%	40.7%
	P(not reported|predicted free)	99.2%		99.2%	99.2%	94.9%		94.9%	94.9%

### Rabies Prediction Model Fit and Parameters

The Spearman correlation between the predicted and reported number of cases was 0.77 in raccoon territory and 0.44 in skunk territory and was applied to the test data sets using the original model (Table 2). Varying the surveillance definitions as described in the *Methods* section did not substantially improve model fit (Table 2), with AUCs that generally varied only by 0.01 or 0.02 across all models.

Within both the skunk and raccoon reservoir territories, counties predicted to have a higher probability of observing rabid animals (if present) were more likely to report rabid bats (AUC 0.76 in raccoon territory and 0.78 in skunk territory).

Reviewing the rabies prediction model parameters, the probability that a county is free from rabies decreases with the increasing number of rabid animals reported in the previous year in the county of interest. The probability decreases with an increasing number of rabid animals reported in the previous year in any adjacent counties, although the latter effect is less impactful. The probability of rabies freedom increases with increasing years since the last rabies case was reported in the county of interest and also increases with increasing years since the last rabies case was reported in any adjacent counties, although again the latter effect is less impactful on the model prediction (model 1A in Table 3).

If a county is not free from rabies, the number of reported cases in 1 year is positively associated with the number of positive reported cases in the following year. The number of cases reported also increases with an increasing number of rabid animals reported in any adjacent counties, although the effect is less strong. Finally, the number of cases reported the next year increases with surveillance points tested over the past 5 years in the county of interest (model 1B in Table 3).

**Table 2:** Performance of original model and models using different surveillance point definitions on the training and validation data sets.

		Spearman correlation predicted: reported cases	AUC^a^ predicted: reported zero cases	AUC predicted free: reported zero cases
**Raccoon model**				
	Original model, training data set	0.82	0.93	0.93
	Different surveillance definitions^b^, training data set	0.81-0.83	0.93-0.93	0.93-0.93
	Original model predictions compared with rabies presence or absence in the next 5 years, training data set	0.89	0.97	0.97
	Original model applied to validation data set (2019)	0.78	0.90	0.90
	Original model applied to validation data set (2020)	0.77	0.90	0.90
**Skunk model**				
	Original model, training data set	0.52	0.89	0.89
	Different surveillance definitions^b^, training data set	0.51-0.52	0.89-0.89	0.89-0.89
	Original model predictions compared with rabies presence or absence in the next 5 years, training data set	0.66	0.88	0.88
	Original model applied to test data set (2019)	0.44	0.91	0.91
	Original model applied to test data set (2020)	0.44	0.91	0.91

^a^AUC: area under the receiver operating characteristic curve.

^b^Explored definition included the following: including only domestic or only reservoir animals, adjusting the relative value of surveillance effort by changing the coefficient applied to domestic animals from 0.1 to 1 times that of reservoir animals, applying specific coefficients to different species based on their observed test positivity rates, changing the number of years of surveillance effort considered from 1 to 8, and normalizing surveillance effort by county size or population.

**Table 3:** Rabies prediction zero-inflated negative binomial model parameters (fit to county-level data on the number of rabid animals detected from 2003 to 2018). Models were fit separately in raccoon reservoir territory and skunk reservoir territory.

	Raccoon territory					Skunk territory				
	Rabies freedom model (1A)		Rabies count model (1B)		Rabies freedom model (1A)				Rabies count model (1B)	
	Value (SE^a^)	*P* value	Value (SE)	*P* value	Value (SE)		*P* value	Value (SE)		*P* value
Intercept (α_0_/β_0_)	−1.22 (N/A^b^)	N/A	−1.18 (N/A)	N/A	−2.00 (N/A)		N/A	−1.46 (N/A)		N/A
α_1_/β_1_: slope log(number of rabid animals reported in the county of interest in the past year + 0.5)	−0.59 (0.08)	<.001	0.38 (0.01)	<.001	−0.51 (0.13)		<.001	0.39 (0.02)		<.001
α_2_/β_2_: slope log(number of rabid animals reported in any neighboring county in the past year + 0.5)	−0.45 (0.06)	<.001	0.22 (0.01)	<.001	−0.40 (0.02)		<.001	0.24 (0.02)		<.001
α_3_: slope log(years with no rabies reported in the county of interest)	1.28 (0.11)	<.001	—^c^	—	1.22 (0.10)		<.001	—		—
α_4_: log(years with no rabies reported in any neighboring county)	0.62 (0.14)	<.001	—	—	0.91 (0.08)		<.001	—		—
β_3_: slope log(surveillance points in the past 5 years + 0.5)	—	—	0.30 (0.01)	<.001	—		—	0.32 (0.01)		<.001
I(r): dispersion parameter	—	—	1.09 (0.03)	<.001	—		—	−0.16 (0.04)		<.001

^a^SE: sensitivity.

^b^N/A: not applicable.

^c^Not available.

### Model-Based Evaluation of Historical Definition

The model predicts an approximately 98% probability of observing zero cases in the next year in both raccoon and skunk territories (Table 4) for a hypothetical county meeting the historical rabies-free definition (ie, 15 surveillance points, 5 years with no cases in the county of interest, and 5 years with no cases in any adjacent counties). This probability is equivalent to the probability of rabies in counties predicted to be rabies free from Table 1, except that Table 1 considers all counties meeting or exceeding the historical rabies-free criteria, whereas Table 4 considers a scenario that exactly meets the criteria.

**Table 4:** Model predictions for a scenario meeting historical rabies freedom criteria. In contrast to this assumes exactly 5 years with no cases reported in the county of interest or any neighboring counties and exactly 15 surveillance points over the past 5 years.

	Model: raccoon territory	Model: skunk territory
NPV^a^: probability of zero cases reported in the next year, historical criteria are met	0.982	0.979
Probability of being rabies free in the next year, historical criteria are met	0.947	0.920
Probability that a case occurs in the next year but goes unreported, historical criteria are met	0.034	0.059

^a^NPV: negative predictive value.

Separating the probability of zeros derived from the rabies freedom model (ie, county is truly nonendemic for rabies) and those derived from the count model (ie, county is endemic for rabies, but no rabies cases are reported), the probability of being truly rabies free is slightly higher for raccoon territory (0.95) than skunk territory (0.92), and the probability that rabies is present but unrecognized is slightly lower in raccoon territory (0.03) than in skunk territory (0.06).

### County-Specific Predictions: Rabies Freedom and Number of Reported Cases

In contrast to the binary yes or no cutoff of the historical rabies freedom definition, the rabies prediction model allows each county to have a predicted probability of being free from rabies and the expected number of terrestrial rabies cases reported in the following year. The median predicted probability of rabies freedom in 2020 was 0.10 (IQR 0.04-0.71) for counties in raccoon territory and 0.91 (IQR 0.55-0.96) for counties in skunk territory. The median expected number of rabies cases reported per county in 2020 was 1.4 (IQR 0.2-3.6) in raccoon territory and 0.03 (IQR 0.01-0.2) in skunk territory. For comparison, the median reported number of rabies cases reported in 2020 was 1.0 (IQR 0.0-3.0) in raccoon territory and 0.0 (IQR 0.0-0.0) in skunk territory. Counties with cases reported in 2020 tended to have a low probability of freedom from rabies (Figure 1).

## Discussion

### Principal Findings

The results of this study show that the historical definition of rabies freedom in the United States is largely successful in identifying counties with the lowest risk of rabies. Applying this definition, 99.2% of counties predicted as rabies free did not have a case reported in the following year. Modeling analyses showed that all 3 components of this definition (in-county presence, neighboring county presence, and surveillance effort) play an important role in making this prediction. Increased number of years since a case was last reported in the county of interest or any contiguous counties is associated with increased probability of rabies freedom, and increased surveillance over the past 5 years is associated with better rabies observation in the subsequent year in endemic counties.

**Figure 2:**
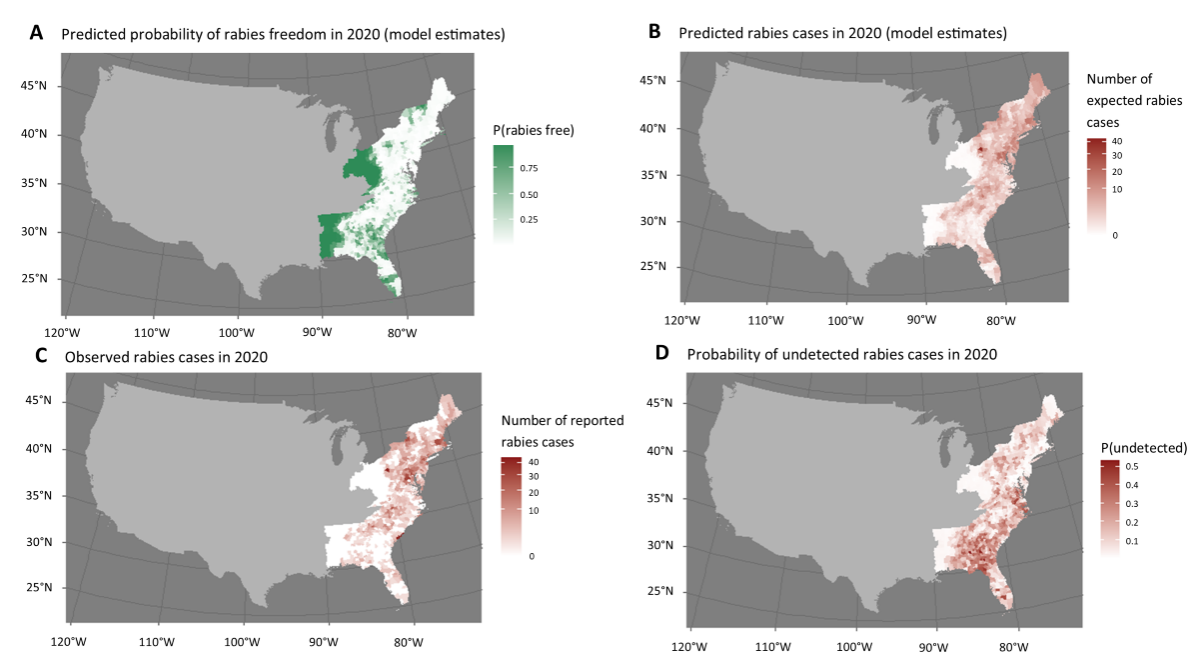
Raccoon territory model predictions of the probability of rabies freedom (A) and the number of expected rabies cases (B), compared with the number of actual reported rabies cases per county (C) in 2020. Model estimated probability that a rabies case will occur but go unreported (D).

**Figure 3:**
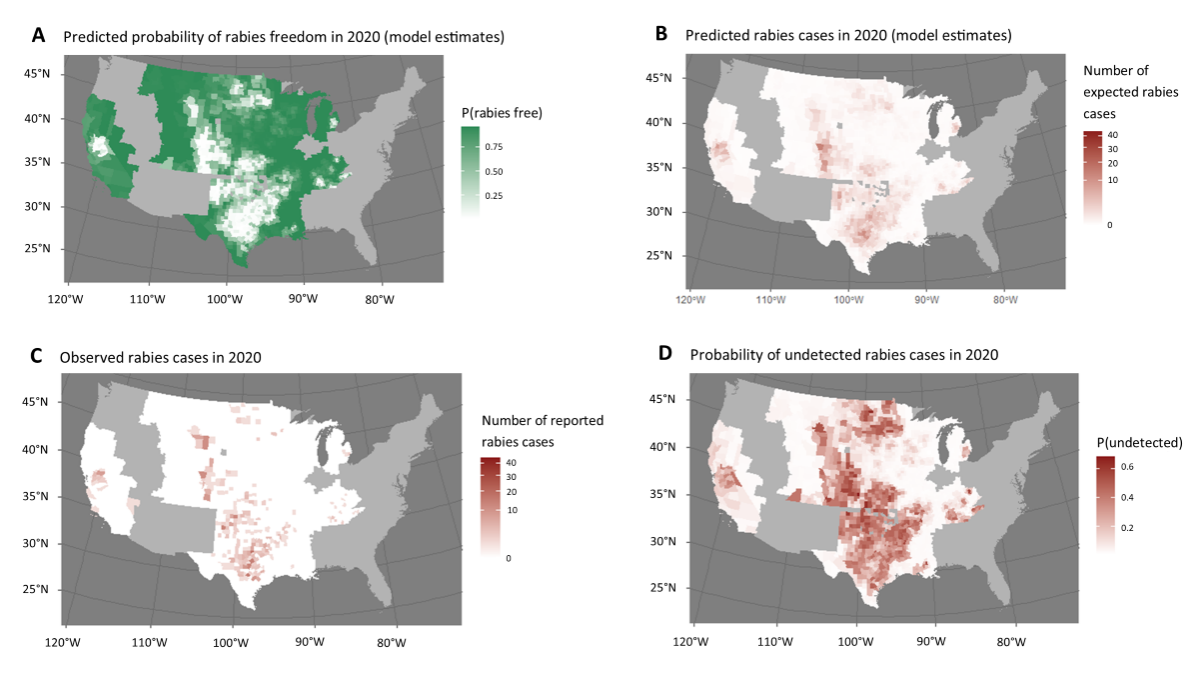
Skunk territory model predictions of the probability of rabies freedom (A) and the number of expected rabies cases (B), compared with the number of actual reported rabies cases per county (C) in 2020. Model estimated probability that a rabies case will occur but go unreported (D).

Although the evaluation method used in these analyses supports the use of the historical definition, it also presents a more nuanced assessment of rabies freedom. Although we did not find an advantage of changing the definition of surveillance points from its historical definition (0.5 points per domestic animal tested and 1 point per reservoir animal tested), the model allowed us to estimate an individualized risk for each county rather than a binary designation of rabies presence or absence (Figures 2 and 3). Predictions of the probability of rabies freedom and the expected number of cases reported in 2019 and 2020 correlated with actual cases reported in the same years.

This analysis only considered variables related to historical rabies surveillance and detection, as in the historical definition. The inclusion of other variables such as county population and the size of land use could be considered in the future. In addition, modifications to these methods that consider the point locations of samples could be considered when subcounty designations are needed. Subcounty location data (eg, Geographic Information Systems coordinates) are not routinely reported to the CDC’s NRSS, necessitating an aggregated county-level analysis. Trade-offs between distance and time from the last case reported could also be considered (eg, is it possible to consider an area “rabies free” if a recent case was reported but is very far from the nearest positive?).

Designations of terrestrial rabies endemicity are important for clinical, public health, and wildlife management decision-making at a local, state, and national scale. The model presented in this study uses data only from the United States, and model parameters may not be generalizable to other countries, particularly those where DMRVVs may be found or where surveillance systems differ substantially from the US NRSS. When considering the application of these methods to locations outside of the United States, several important considerations must be made. First, the models described here differed according to the reservoir species. This suggests that a modeling process is required for each unique rabies reservoir species, of which >30 species have been described globally. Second, administrative divisions may differ substantially from US counties by size or population, making rabies detection more or less likely.

The United States has one of the most robust rabies surveillance systems in the world, with more laboratories and testing per capita than nearly any other country. From 2016 to 2020, nearly all counties in the United States tested at least 1 terrestrial animal for rabies (Figure 1). Therefore, under the US system, the amount of testing conducted in a county-year may not be as important as having access to laboratories and relatively high rabies awareness among the public and health jurisdictions. According to the US surveillance scheme, the cost of testing and access to laboratories are rarely cited as reasons for not testing animals suspected of having rabies. Although access to rabies testing in the United States is robust, 1 sensitivity analysis presented in this study showed that counties predicted to detect more terrestrial rabies cases (ie, those with greater surveillance efforts for terrestrial animals and more terrestrial animal rabies reported in the past year) are more likely to detect rabid bats in the following year. This could indicate that accessing testing is not equivalent in all counties, and future analyses could consider investigating ways to identify locations where a lack of testing could overshadow rabies virus transmission. In countries where access to testing is less secure and awareness of rabies and appropriate postexposure behaviors is lacking, predictive variables (such as number of samples tested) are likely to be more important in predicting the presence or absence of rabies in a defined area.

### Limitations

This study had several limitations. Variant typing was not performed on all rabid animals in the United States, and some rabid animals with bat variants may have been inadvertently included in our data set. However, previous studies have found that >99% of terrestrial mammals in an area with a terrestrial RVV have the expected viral variant [26]. Of the 9 county-years for which rabies was reported despite meeting the rabies freedom definition, at least 2 had bat variants that were not reported to the CDC at the time of the analysis. Some states inconsistently reported negative rabies testing results in the early years of our data, leading us to exclude certain years of data from some states; however, <10% of the data were affected. In addition, we included data from the United States only, so counties bordering Mexico or Canada have incomplete data on rabies cases in the surrounding areas. We only explicitly developed models for raccoon and skunk RVV territories, as the number of counties with other terrestrial rabies variants in the United States is small. Counties in states, such as Arizona and New Mexico, with multiple RVVs were not included when fitting the models, as the rabies dynamics may differ in these regions. It is possible that animal location data submitted to the NRSS may not reflect the location where the animal was exposed to rabies but could reflect other locations noted by case investigators such as the location where the animal was found. Finally, it is possible that even counties in which no rabies is reported over long periods may not truly be free from rabies.

### Conclusions

In conclusion, the historical rabies freedom definition is a reasonable approach for identifying counties that are truly free from terrestrial raccoon and skunk rabies virus transmission. Gradations in risk can also be measured using the rabies prediction model presented in this study, with AUCs >0.9. Nevertheless, bat variant rabies in terrestrial mammals remains a possibility throughout the United States, excluding Hawaii, and translocation events can occur, which can lead to unpredictable shifts in rabies epidemiology [16]. Even counties with a high probability of rabies freedom should maintain vigilance and rabies testing capacity, as there are numerous examples of anthropogenic and natural translocations of rabies-infected animals that can cause major changes in the epidemiology of rabies.

## Abbreviations

AUC: area under the receiver operating characteristic curve
CDC: Centers for Disease Control and Prevention
DMRVV: dog-mediated rabies virus variant
NRSS: National Rabies Surveillance System
PEP: postexposure prophylaxis
RVV: rabies virus variant

## Data Availability

The data sets generated and analyzed during this study are available from the corresponding author upon reasonable request.
